# A whole lung in silico model to estimate age dependent particle dosimetry

**DOI:** 10.1038/s41598-021-90509-8

**Published:** 2021-05-27

**Authors:** Kamran Poorbahrami, Irene E. Vignon-Clementel, Shawn C. Shadden, Jessica M. Oakes

**Affiliations:** 1 Department of Mechanical and Industrial Engineering, Northeastern University, Boston, USA; 2 Inria Saclay-Ile-de-France, Inria, France; 3 Department of Mechanical Engineering, University of California Berkeley, Berkeley, USA; 4 Department of Bioengineering, Northeastern University, Boston, USA

**Keywords:** Paediatrics, Drug delivery, Biomedical engineering

## Abstract

Anatomical and physiological changes alter airflow characteristics and aerosol distribution in the developing lung. Correlation between age and aerosol dosimetry is needed, specifically because youth are more susceptible to medication side effects. In this study, we estimate aerosol dosages (particle diameters of 1, 3, and 5 $\upmu$m) in a 3 month-old infant, a 6 year-old child, and a 36 year-old adult by performing whole lung subject-specific particle simulations throughout respiration. For 3 $\upmu$m diameter particles we estimate total deposition as 88, 73, and $66\%$ and the conducting versus respiratory deposition ratios as 4.0, 0.5, and 0.4 for the infant, child, and adult, respectively. Due to their lower tidal volumes and functional residual capacities the deposited mass is smaller while the tissue concentrations are larger in the infant and child subjects, compared to the adult. Furthermore, we find that dose cannot be predicted by simply scaling by tidal volumes. These results highlight the need for additional clinical and computational studies that investigate the efficiency of treatment, while optimizing dosage levels in order to alleviate side effects, in youth.

## Introduction

Aerosol medications are frequently used to treat respiratory diseases in children and infants, yet the relationship between age and dose remains unclear. Most inhaled therapeutics are designed for children 4 years and older, requiring clinicians to prescribe medications off label for treatment of respiratory diseases in infants^1^. On the other hand, while it is evident that children are more susceptible to toxic particles^2,3^, it is not well understood how this risk changes throughout the lifespan. The pulmonary airways continue to develop throughout postnatal growth, impacting both the structure and function of the respiratory tract^4^, leading to changes in regional and total dosimetry. Indeed, the fate of inhaled particles in the lung is a function of anatomy, minute volumes, and particle size/shape. Depending on the deposition location, size, and material composition, aerosol particles may be leveraged to treat disease or cause acute and/or chronic health consequences.

The advantage of inhaled aerosol medications is that the drugs can be delivered directly to the sites being treated and are therefore an ideal option for respiratory disease treatment (e.g., asthma, cystic fibrosis, respiratory infections). In addition, because of their potential to enter the systemic circulation through the vast pulmonary capillary network, inhaled therapeutics are now being considered as an effective way to deliver vaccinations^5^ and gene therapies^6,7^. While inhaled medications can provide quick relief of symptoms, care must be taken to provide adequate dosage levels to avoid adverse acute and chronic side effects (e.g. dysphonia, impaired growth in children, decreased bone mineral density, etc.)^8,9^, especially for infants^1^. Therefore the key to aerosol therapy management, across all age groups, is to prescribe dose levels that effectively treat the disease while reducing the side effects.

Toxic aerosols, on the other hand, may cause an inflammatory cascade, impacting the respiratory^10,11^ and cardiovascular systems^12,13^, as well as other peripheral organs and systems^14^. Evidence via epidemiological^15^ and modeling studies^16^ suggests that children are more susceptible to airborne toxins than adults. This is likely because they are still developing, as discussed in the review paper by Ginsberg et al.^17^. Compared to the adult lung, juveniles have smaller airway dimensions and fewer alveoli. In addition, their alveolar structures are not as well defined (particularly for infants^18^), and they inhale greater amounts of air relative to their lung volume and body weight^19,20^. Therefore, public health policies regarding inhalation exposure should consider the state of post-natal lung development when deciding on exposure concentration limits.

As suggested by others^21–24^, anatomical and physiological differences between youth and adults likely result in enhanced dosimetry in juveniles, however the disparity in both the total and regional particle deposition locations between age groups has been relatively unexplored. The vast majority of devices and drugs prescribed for infants and children are similar to the ones developed for adults, due to the lack of clinical data to suggest alternative designs^1^. Performing clinical trials on otherwise healthy children is often not feasible or ethical and therefore modeling approaches may be employed, such as use of animal^25^ and non-human primate models^26,27^, bench-top *in vitro* models^19,28^, or computational simulations^29^, each having their advantages and disadvantages.

Computer models of aerosol dosimetry can supplement or alleviate the need to perform extensive experimental studies, especially in susceptible populations. Previous modeling studies of aerosol dosimetry in children have either incorporated the entire lung by use of a representative single-path model^24^ or have selected specific regions of the respiratory system (e.g extra-thoracic airways^30^, a portion of the conducting airways^31^, or a representative acinar region^18^) to study. Recently, Das et al.^29^ simulated airflow and particle transport throughout inhalation in idealized self-similar upper airway geometries of 5, 10, and 25 year old subjects. This previous study highlighted similarities in flow distributions and deposition fractions between all ages, when Stokes number was the same between subjects. Furthermore, Das et al.^29^ found that smaller particles are needed to optimize delivered dose to the conducting airways in children, compared to adults. In contrast to region-specific models, single-path methods (grouping all airways within a given generation) may enable total, lobar, or generation-based dosimetry to be assessed. However single-path methods are unable to uncover specific deposited particle locations (e.g. hotspots), do not account for inter-subject variability in airway dimensions, and cannot incorporate the complex flow patterns that exist within the pulmonary airways. Alternatively, image-based patient-specific models enable identification of hotspots, however they are unable to provide total and lobar level dosimetry measures. To overcome individual modeling limitations, we recently created a framework that couples 3D image-based models with 1D lower-dimensional models, enabling predictions of both regional and total deposited particle concentrations throughout the respiration cycle^32,33^.

The main goals of this study are to comparatively predict dosimetry in three subject-specific models: infant, child, and adult. To accomplish this goal, we perform coupled simulations, incorporating CT-based airway geometries, realistic respiratory wave forms^34^, and particles with diameters that represent aerosol medications that are currently on the market (1, 3, and 5 $\upmu$m). Model outcomes highlight regional differences at both the lobar and regional (conducting versus respiratory) levels as a function of particle size and subject age. By coupling the central and distal airways together, we compare localized central airway deposition hotspots between the age groups for both inspiration and expiration. With these model predictions we present area-normalized dosage levels, motivating follow-up studies that aim to optimize aerosol treatment strategies, enabling patient size and/or age to be accounted for.

## Materials and methods

**Figure 1:**
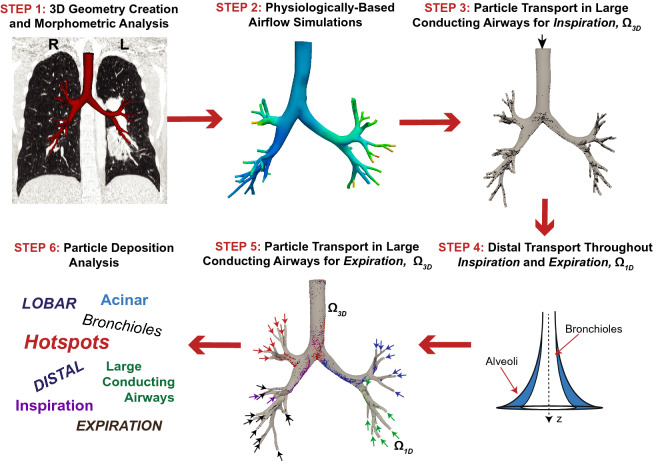
Simulation pipeline for the multi-dimensional airflow and transport simulations. Airway geometries were previously created from CT images and airway morphometry was measured from the geometric models (Step 1)^34^. In Oakes et al.^34^ airflow was simulated throughout the respiration cycle by a pressure differential that overcame the respiratory resistance and compliance to drive air in and out of the lungs (Step 2). Particles are then tracked throughout the respiration cycle by first calculating their individual trajectories in the 3D models for inspiration (Step 3). Next, the aerosol bolus is convected through the distal regions of the lung by solving reduced-order models with a deposition loss term (Step 4). Finally, the particle trajectories are solved throughout expiration in the 3D airways (Step 5) and the regional deposition patterns are assessed (Step 6).

Particle predictions in representative infant, child, and adult airways are performed by using a combination of airflow and particle transport computational solvers which incorporates physiologically realistic respiration and CT-based anatomy (Fig. 1). Patient-specific airway geometries of a 3 month, 6 year, and 36 year old subjects (demographics provided in Table 1) were previously created from clinically-obtained CT scans. The Institutional Review Board at Stanford University approved the study protocol and all research was performed in accordance with relevant regulations. As the study was retrospective, the Stanford University IRB granted waiver of informed consent. Airway morphometric features of these subjects were characterized and compared to individuals within the same age range^34^. Airflow velocities were then calculated in the 3D models with computational fluid dynamic simulations^35^, where airflow was driven by a pressure differential, incorporating the distal respiratory resistance and compliance^34^. These previous efforts set the stage to investigate particle dosimetry within these patient-specific airways.

With the aim of predicting both localized and regional particle fate in the lungs, we employed a multi-domain method which we previously developed^32^, enabling us to quantify particle fate in the entire lung and throughout a respiration cycle. As all airways could not be identified from clinically-obtained CT scans, it is impossible to create a 3D realistic geometry of the whole lung. To overcome this limitation, we couple the 3D subject-specific geometries ($\Omega _{3D}$) to generic models that incorporate the distal airways that cannot be imaged ($\Omega _{1D}$). Particle transport throughout a respiration cycle (inspiration and expiration) are calculated by connecting the $\Omega _{3D}$ and $\Omega _{1D}$ models together (Fig. 1).

**Table 1:** Subject demographics and estimated^36,37^ (in bold) or calculated (in italicized bold) respiration parameters.

	Age (years)	Weight (kg)	Height (cm)	Sex	TV (mL)	$\frac{TV}{TV_A}$	$V_{FRC}$ (mL)	RR (breaths/s)	$\mathbf{A}_{\mathbf{FRC}}$ (m$^2$)	$\mathbf{BSA}$ (m$^{2}$)
Infant	0.25	6.1	55.2	Female	***49***	*0.10*	***81***	**0.66**	**3.2**	**0.31**
Child	6	18.8	115	Male	***209***	*0.42*	***830***	**0.32**	**27.4**	**0.77**
Adult	36	59.8	160	Female	***500***	*1*	***3000***	**0.25**	**64.7**	**1.64**

*TV* tidal volume, $\frac{TV}{TV_A}$ tidal volume with respect to adult’s tidal volume, $V_{FRC}$ FRC volume, *RR* respiratory rate, $A_{FRC}$ total surface area at FRC, *BSA* total body surface area.

On average 9 million rigid spherical particles ($d_p=1,3,5$ $\upmu$m, $\rho _p=1$ g/ml) are released throughout inhalation, seeded uniformly at the trachea inlet. The particle seeding rate is prescribed such that the released particle concentration is proportional to the local flow rate of the element that they originate from^32,38^. The trajectory of individual particles is calculated within the 3D airways based on a momentum balance that accounts for both drag and gravitational forces^32,39^. Particles will deposit on the airway walls when the distance between the center of the particle and the airway wall becomes less than or equal to its radius. In contrast, the collective aerosol bolus (e.g. aerosol concentration) is simulated in the distal airways ($\Omega _{1D})$. Therefore, to pass particles between the 3D and 1D regions during inspiration we need to change the description of the particles from tracking individual particles to particle concentration and vice-versa during expiration. Therefore, the number of particles re-entering is calculated based on the particle concentration exiting the segment-specific 1D regions. By doing this, we account for the number of particles that deposit distal to the 3D model when seeding particles throughout respiration.

The aerosol bolus is calculated in $\Omega _{1D}$ by employing an adapted form of the well-known trumpet model^32,40^. In general, particle concentrations ($\frac{\mu g}{mL}$) are translated through $\Omega _{1D}$ by a combination of mixing (e.g. diffusive) and advective transportation. Here, the mixing term accounts for heterogeneity in the branching structure and merging of airways; the mixing term is a function of the standard deviation of the airway length and the conduit air speed^32^. The advection term, which dominates the bolus movement for particle sizes considered here, incorporates the transportation of particles convected by the inhaled air. Particle loss to the airway walls is modeled with a loss term, incorporating empirical formulas representing deposition due to gravitational, drag, and diffusive forces^32^.

### Distal airways

As the resolution of thoracic CT images limits our ability to create airway geometries that span the entire lung, we must employ alternative methods to describe all airways downstream of the 3D geometry. To overcome this challenge, we obtain generation-based airway dimensions of similar-aged subjects from the literature^41^. These empirical formulas describe the diameters and lengths as a function of their generation and lobe. Note, the airway generation number successively increases at each bifurcation. Airway dimensions were scaled to match the functional residual volume ($V_{FRC}$, Table 1) of a similar aged-subject.

### Connecting the 3D patient-specific geometry to the generic distal airways

Each distal airway of the 3D model is connected to the $\Omega _{1D}$, where the $\Omega _{1D}$ represents all airways distal to that particular airway. To merge the two models ($\Omega _{3D}$ and $\Omega _{1D}$), we choose to match the cross-sectional areas at the interface between the two domains. When connecting the airways, we ensured that the FRC volume of each lobe matched the expected values using subtended lobe volume fractions (0.25, 0.20, 0.25, 0.09, 0.21, for the left inferior and superior, and right inferior, middle, and superior lobes, respectively)^34,42^.

### Post processing

**Figure 2:**
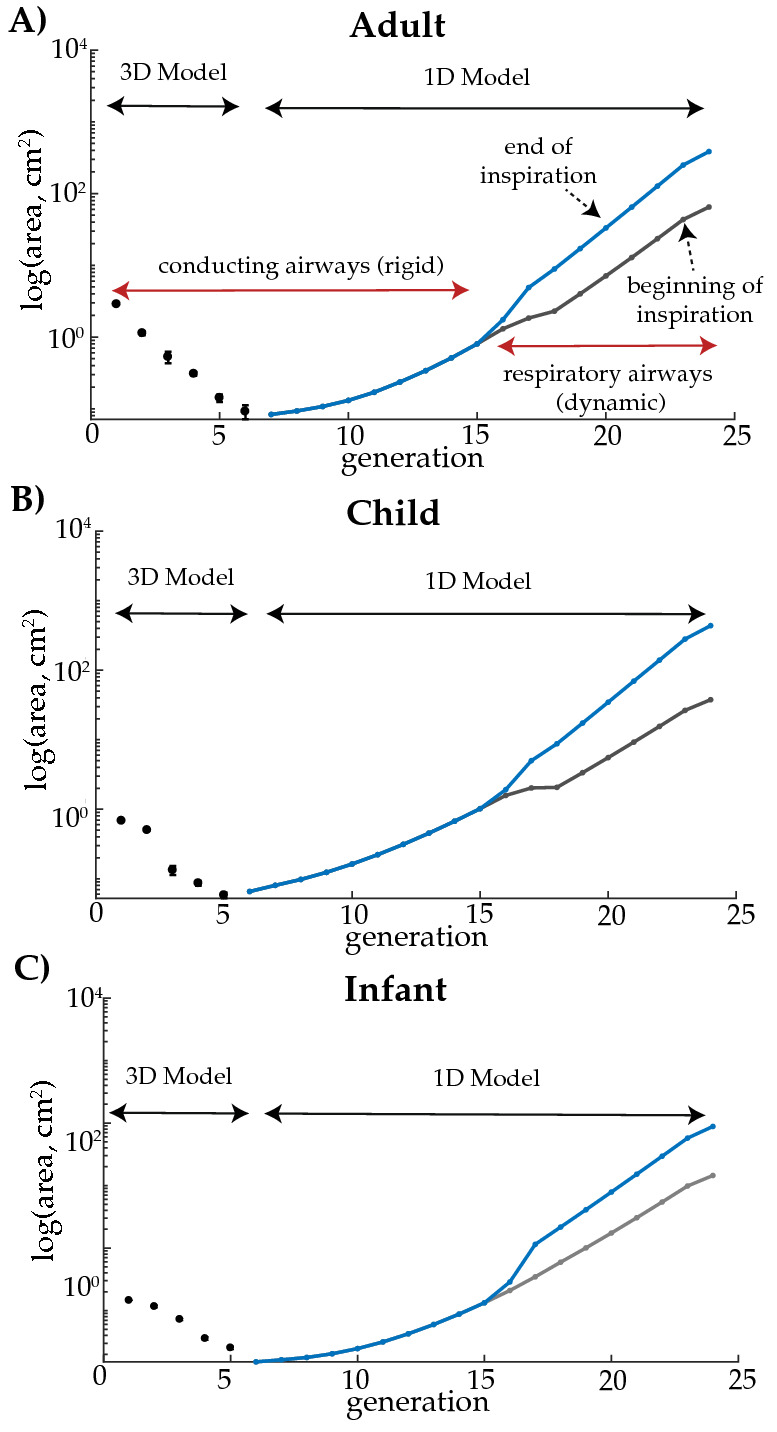
Cross-sectional areas of representative branches in the lower left lobe of the adult (panel **A**), child (panel **B**), and infant (panel **C**) models. Note, for the outlined 3D regions, the areas are measured directly from the image-based models (error bars represent the standard deviation of the airways within each generation). The 1D regions are idealized and scaled to match FRC (Table 2). The conducting and respiratory zones are highlighted in panel (**A**), where the blue and gray lines represent the total cross-sectional area at the end and beginning of inspiration, respectively.

Total deposition percentages ($Dep_T$) are found by dividing the number of deposited particles by the total particles inhaled (unless stated otherwise). Conducting versus respiratory airway deposition ratios (CR ratio) are calculated by dividing the number of particles deposited in the conducting zone (C) by the number of deposited particles in the respiratory zone (R). Conducting and respiratory zones are highlighted in panel A, Fig. 2. Regional deposition (e.g., lobar, C and R zones) concentrations are calculated by dividing the number of deposited particles in each region by the total surface area of that region. We calculate the area-normalized number of deposited particles during the whole breathing cycle ($NP=\frac{N_e \sum _T}{N_T \sum _e}$, where $N_e$ is the total number of deposited particles (for both inhalation and exhalation) within each surface element, $\sum _T$ is the total surface area of the 3D geometry, $N_T$ is the total number of deposited particles, and $\sum _e$ is the element’s surface area). Note, that we increased the mesh dimensions for visualization purposes only. The mesh cell size is similar between the subjects to enable direct comparison of *NP*.

## Results

### Airway geometry

Cross-sectional areas, presented as a function of airway generation, are shown in Fig. 2 for a representative branch within the left inferior lobe for the adult (panel A), child (panel B), and infant (panel C). The image-based 3D region ($\Omega _{3D}$) and the idealized 1D airways ($\Omega _{1D}$) are outlined. The 1D models represents the total cross-sectional area of all the branches within each generation distal to the representative 3D airway. The respiratory zone of the lung begins at generation 16 for the lower left lobe of the child and adult models and 17 for the infant model (Fig. 2). As highlighted in Fig. 2, the respiratory airways expand and contract throughout the respiration cycle to accept and release the inhaled air. Lobe-specific morphometric dimensions employed in the 1D models, scaled to match expected FRCs (Table 2), are provided within the supplementary materials.

### Deposition throughout the lung

**Figure 3:**
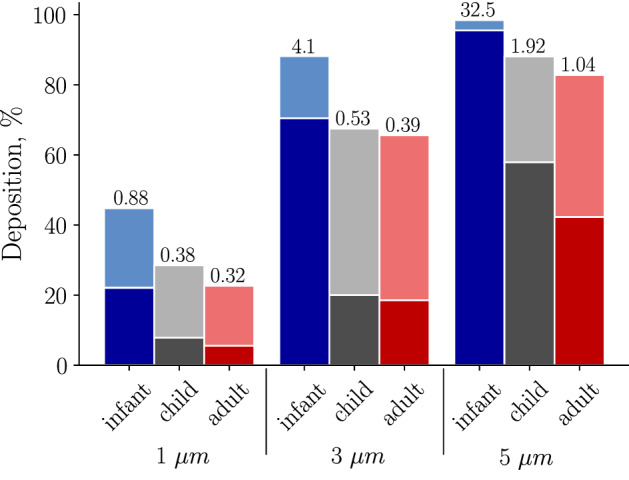
Whole lung deposition percentages for the three models are shown for each of the particle sizes simulated. Note, the conducting (*C*) and respiratory (*R*) zones are shown in dark and light shades, respectively. The ratio of deposited particles between the conducting and respiratory zones, CR ratio, are displayed above each bar. Figure 2 points to the conducting and respiratory zones. Note, the conducting zone includes both the 3D model and part of the 1D model.

In Table 2, we present total deposition percentages ($Dep_T$) as well as several scaling parameters to take into account differences in TV and body size. Deposition percentages ($Dep_T$) are largest in the infant, compared to the adult and child subjects (Table 2); $Dep_T$ increases with particle size for all age groups. While the majority of the 3 and 5 $\upmu$m diameter particles deposit in the lung, most of the 1 $\upmu$m particles are exhaled back into the environment (Fig. 3). The $\frac{Dep_T \times TV}{A_{FRC}}$ parameter (Table 2) may be multiplied by the inhaler drug concentration (e.g., $\frac{\mu g}{mL}$) to obtain a deposited dose (mass of the deposited drug relative to the resting lung surface area, $A_{FRC}$, $\frac{\mu g}{cm^2}$). Therefore, for an equivalent drug concentration the infant and child subject will have larger deposited dose compared to an adult. Deposition scaled by tidal volume ratio ($Dep_T \times \frac{TV}{TV_A}$, Table 2) highlights that although deposition fraction is highest in the infant, the infant inhales a much smaller air volume and therefore the infant receives less total mass in comparison to the child and adult models for a single breath. We also calculate the deposition rate ($Dep_T\times TV\times RR$, Table 2) which can be used to predict deposited dose of particulate matter in human lungs by multiplying this variable by the particulate matter concentration and exposure time, valuable for toxicology studies. In addition, we normalize that variable by the body surface area (*BSA*) to show that for a given exposure time, infants will inhale less particulate matter; however, the dose relative to their BSA is higher.

**Table 2:** Calculated whole lung dosimetry for the three age groups and particle sizes.

	$d_p$ $\upmu$m	$Dep_{T}$ $\%$	$\frac{Dep_T \times TV}{A_{FRC}}$ $\%-mL/cm^2$	$Dep_T\times TV\times RR$ $\%-L/s$	$\frac{Dep_T}{BSA}$ $\%-L/s-m^2$	${Dep_{T}}\times {\frac{TV}{TV_A}}$ $\%$	$\frac{Dep_C}{A_C}$ $\% / cm^2$	$\frac{Dep_R}{A_R}$ $\% / cm^2$
Infant	1	44.9	0.070	1.45	4.68	4.5	4.40E−2	77E−5
	3	87.1	0.135	2.82	9.10	8.7	15.0E−2	55E−5
	5	98.5	0.153	3.19	10.29	9.9	20.0E−2	7.6E−5
Child	1	28.5	0.022	1.91	2.48	12.0	0.35E−2	7.6E−5
	3	72.5	0.055	4.85	6.26	30.5	1.10E−2	17E−5
	5	88.1	0.067	5.89	7.65	37.0	2.60E−2	11E−5
Adult	1	22.6	0.017	2.83	1.73	22.6	0.11E−2	2.7E−5
	3	65.6	0.051	8.20	5.01	65.6	0.39E−2	7.3E−5
	5	82.9	0.064	10.36	6.32	82.9	0.88E−2	6.3E−5

$d_p$ particle diameter, $Dep_{T}$ total deposition (inspiration and expiration together), total tissue dose ($\frac{Dep_T\times TV}{A_{FRC}}$*:* where TV is the tidal volume and $A_{FRC}$ is the lung surface area at FRC, Table 1), rate of deposited particulate matter ($Dep_T\times TV\times RR$: where *RR* is the respiratory rate, Table 1), normalized by body surface area, *BSA:* $\frac{Dep_T\times TV\times RR}{BSA}$, relative deposition percentages compared to the adult (${Dep_{T}}\times {\frac{TV}{TV_A}}$), conducting region tissue dose ($\frac{Dep_C}{A_C}$), and respiratory region tissue dose ($\frac{Dep_R}{A_R}$).

### Central and peripheral deposition

Whole lung deposition percentages are shown for the three subjects and 3 different particle sizes (1, 3, and 5 $\upmu$m diameter, Fig. 3). Largest deposition fractions are found in the infant due to the relatively smaller airway sizes and higher airflow speeds^34^. In addition, the infant has the highest conducting versus respiratory deposition ratio (CR ratio) for all particle sizes. Larger particles deposit more in the conducting airways due to inertia as highlighted by the CR ratio (when $\hbox {CR}>1$ there is more central deposition, while $\hbox {CR}<1$ indicates more peripheral deposition). Note, that in the infant, most of the particles are filtered in the conducting airways during inhalation; only a few reach the peripheral region and have the chance to deposit distally. In addition the infant showed higher tissue dosages in both in the conducting and respiratory regions ($\frac{Dep_C}{A_C}$ and $\frac{Dep_R}{A_R}$, respectively) due to their smaller lung surface area (Table 2).

### Region-specific deposition

**Figure 4:**
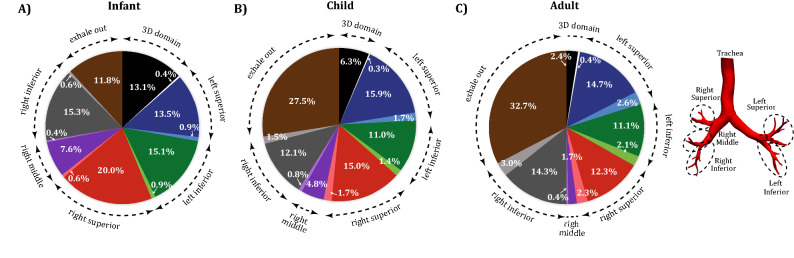
Whole lung deposition percentages (for 3 $\upmu$m diameter particles) for the infant (**A**), child (**B**), and adult (**C**) airways. The 3D geometry as well as the five lobes are highlighted, with expiration represented with the lighter shades. The percentage of particles exhaled out of the lungs are also shown for each of the age groups.

Fewer particles deposit during exhalation than during inhalation, as shown in Fig. 4 for $3\mu \hbox {m}$ diameter particles. Similar deposition percentages are seen for the left superior and right superior lobes in the child and adult models; particles preferentially deposit more in the right superior lobe in the infant model. Deposition percentage in the 3D geometry is reversely correlated with age. Only 11.8% of the $3\mu m$ particles are exhaled from the infant, whereas 27.5% and 32.7% of the particles are exhaled back into the environment from the child and adult models, respectively (Fig. 4).

### Central airway deposition

**Figure 5:**
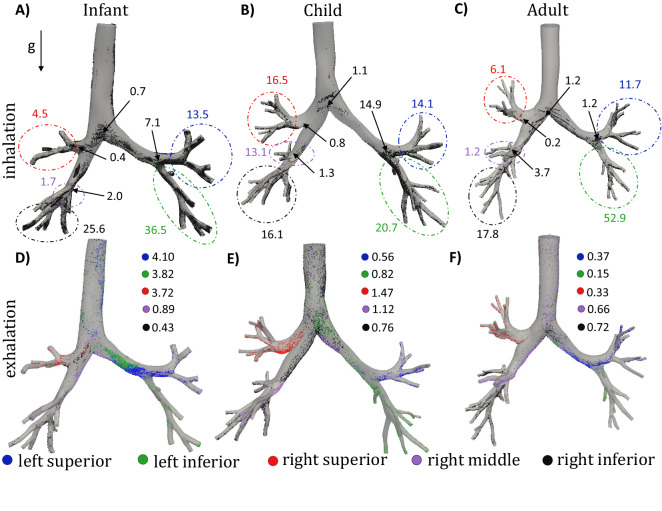
Deposited particle locations for infant (panels **A**,**D**), child (panels **B**,**E**), and adult (panels **C**,**F**) for the 3 $\upmu$m diameter particles. Panels (**A**–**C**) highlight deposited hotspots for inspiration, where the values represent the regional deposition percentage (based on the number of particles depositing during inhalation only). Hotspots for expiration are shown in panels (**D**–**F**); the particles are color coded based on the lobe that they originated from. Presented values (panels **D**–**F**) are the percent of deposited particles, calculated from the number of particles released back into the 3D model (not total deposition percentages).

Particle deposition (3 $\mu \hbox {m}$) locations for each subjects are shown in Fig. 5 at the end of inhalation (first row, showing both lobar and main bifurcations deposition percentages) and at the end of exhalation (bottom row). Note, the numbers presented in the first row are showing the regional deposition with respect to the total deposition within the 3D model during inhalation. High variability can be seen between and within all subjects. For example, 37% of the deposited particles landed on the 3D portion of the left inferior lobe of the infant subject, while deposition in the child and adult models are 21% and 53%, respectively. Numbers in Fig. 5D–F show the percentage of particles that deposited within the 3D geometry with respect to the total particles entering the model throughout each lobe during exhalation. For instance, only 4.1% of the particles that re-entered the 3D geometry during exhalation deposited within the 3D geometry. As highlighted in Fig. 5 most of the particles deposit in the direction of gravity and near the bifurcations regions during inspiration. In contrast, we observe regions of high deposition concentrations downstream of the bifurcation regions (relative to the flow direction), where two daughter branches feed into the parent branch during exhalation. Particle deposition during exhalation and its correlation with the flow field (e.g. helicity^33^ and wall shear stress divergence^43^) has been shown previously in healthy adults.

A substantial amount of particles deposit at the bifurcation regions located at the junction between the inferior and superior left lobe, especially for the child (14.9%), and less so for the infant (7.1%). These percentages are relative to the percent deposition in the 3D model for inhalation. Deposition for this carina is minimal for the adult, with the most deposition occurring at the bifurcation leading to the right middle lobe (3.7%). Note, the bifurcation angles (angle between the two daughter branches) are: $61^{\circ }$, $73^{\circ }$, and $83^{\circ }$ and the daughter to parent diameters area ratios are: 0.67, 0.78, and 0.77 for the infant, child, and adult, respectively.

**Figure 6:**
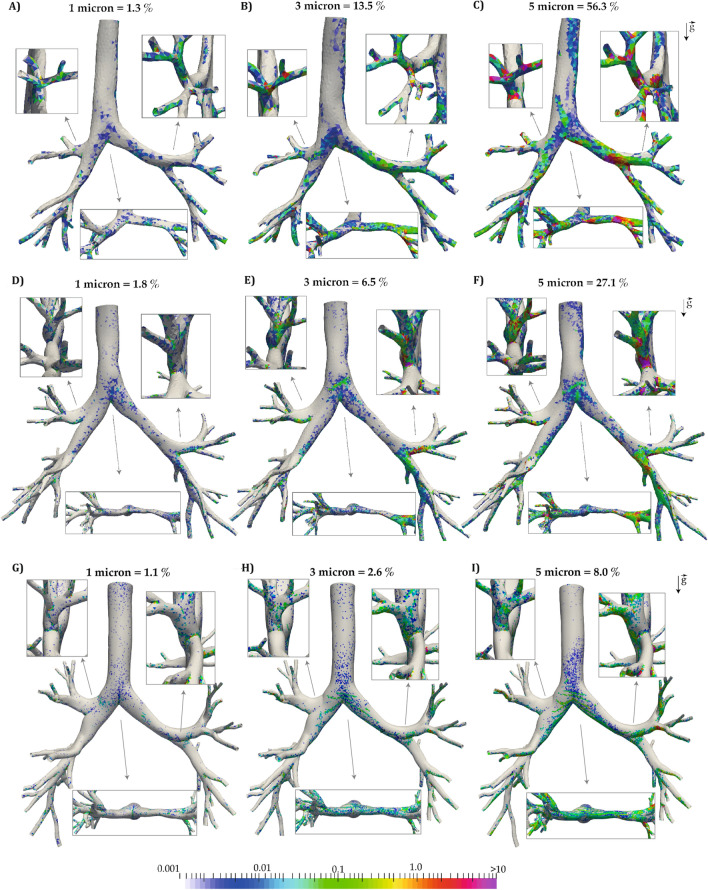
Deposited particle element concentrations ($NP=\frac{N_e \sum _T}{N_T \sum _e}$) for the infant model (panel **A**–**C**), for the child model (panel **D**–**F**), and for the adult model (panel **G**–**I**) for $d_p =$ 1 $\upmu$m, 3 $\upmu$m, and 5 $\upmu$m (inspiration and expiration are plotted together). NP is unit-less.

Element concentrations (*NP*) for inhalation and exhalation together are shown for the three particle sizes for the infant, child, and adult in Fig. 6. Larger values of *NP* indicate more deposition relative to the element’s surface area. These figures show a more quantitative measure of deposition hotspots than in Fig. 5 (exhalation and inhalation together) for all sizes of particles and highlight that deposition concentration is highest at the bifurcation regions for all three sizes of particles and the three subjects. Particle concentrations are higher on the left inferior lobe of the child as showed in Fig. 6E and supported by Fig. 5B. Particles typically deposit in the gravitational direction, particularly for the larger particle diameters as shown in the inset figures. Regions of high deposition concentration ($NP > 10$) cover a relatively larger surface area in the infant compared to the other subjects. Deposited lobar concentrations on $\Omega _{3D}$ for each particle size is provided within the supplementary materials.

## Discussion

Despite the fact that infants and children are more susceptible to aerosol particles than adults, there are few studies that focus on total and regional particle deposition in their lungs. The majority of prescribed inhaler devices and drug formulations are designed based on experimental studies in adults or older children^1,18,23^. However, young children and infants have lungs that are smaller than their adult counterparts, with the size depending on the age, height, and sex of the child^44^. As shown in Oakes et al.^34^ the small airway dimensions, compared to adult lungs (trachea cross sectional area of the infant is $7\%$ of the adult lung), lead to faster airflow speeds through their airways, which will impact total and regional particle deposition.

Our results demonstrate for the first time the influence of lung size in total and regional deposition both in the conducting and peripheral regions throughout a respiration cycle. Deposition efficiency is highest in the infant lung (Fig. 3) in comparison to the child and adult, whatever the particle size. This is mainly caused by their greater fraction of tidal volume with respect to FRC, along with their smaller airways, which leads to faster flow speeds^34^. Both the deposition % and the CR ratio increase non-linearly with particle size for all age groups (Fig. 3). Moreover as particle size increases, there is a switch from respiratory zone dominated deposition to conducting airway dominated deposition. However, this switch occurs at different particle sizes based on the age group. More particles are filtered (e.g. deposit within the large conducting airways and respiratory regions) during inhalation in infants, resulting in less deposition throughout exhalation, in comparison to the child and adult models (Fig. 4). Here, we only have one subject in each group: more studies in this area are needed to find the variability between subjects in each age-group.

Children’s lungs are growing and their innate defence to the drugs side effects may be impaired. Due to the limited number of clinical trials in infants and children, it remains challenging to find the best inhaler for this population along with the drug characteristics such as dose and particle size^18,45^. Therefore, patients within this age-group need to be followed more closely by clinicians to monitor the outcomes of their treatment^1^. Here we calculate total lung deposition efficiency ($Dep_T$, percent of the inhaled particles that deposited) and examine how $Dep_T$ compares across the three age groups by multiplying $Dep_T$ by the tidal volume relative to an adult’s TV ($\frac{TV}{TV_A}$, a suitable measure for a single breath). While the deposition efficiency is higher in the infant and child, compared to the adult (Table 2), the tidal volume is several times lower (Table 1), resulting in much smaller $Dep_T\times \frac{TV}{TV_A}$. This highlights that deposition efficiencies, or deposited masses, are not just a scaling fraction of tidal volume and cannot be simply predicted based on TV alone. This is further shown by deposited tissue dose concentrations, which may be calculated by multiplying $\frac{Dep_T\times TV}{A_{FRC}}$ by a known drug concentration. This parameter highlights that the amount of drug relative to the lung surface area is inversely correlated with age (Table 2). Indeed, for a single breath the infant and child inhale larger volumes relative to the surface area ($A_{FRC}$) of the lung (Table 1), resulting in larger deposited tissue dosages. Based on this and future studies, we speculate that respiratory therapists and/or physicians may ultimately be able to estimate the dosage levels for children and infants in order to obtain needed tissue concentrations by multiplying by this factor ($\frac{Dep_T\times TV}{A_{FRC}}$). On a more immediate term, we can provide by future sensitivity analysis of this model, quantitative information about how concentrations and doses change by varying patient versus drug characteristics.

While therapeutics are typically delivered with a single puff, exposure to pollution is cumulative over a period of time. Thus, the rate of deposited dose ($Dep_T\times TV\times RR$) is useful, where the total deposited mass may be determined by multiplying this parameter by the exposure concentration and duration. We observe that while deposition efficiency is higher in the infant and child subjects, compared to the adult subject, the rate of deposited dose is smaller (Table 2). Therefore, for the same exposure concentration and duration, youth will receive less deposited mass. However, relative to their *BSA*, the exposure may be 2.7 times greater in an infant, compared to an adult.

We did not take into account the effects of extra-thoracic airways, mainly because of imaging restrictions. Indeed, incorporating the extra-thoracic airways will result in fewer particles reaching the conducting airways^30^. To address the impact of this assumption, we utilized predictive equations to estimate mouth-throat deposition in the adult and child subjects and nose-throat deposition in the infant subject (as infants are primarily nasal breathers). For the adult and child subjects we utilize the equations provided by Grgic et al.^46^; Grgic et al. used gamma scintigraphy and gravimetry to measure particle deposition of $d_p =$ 3-6.5 $\upmu \hbox {m}$ in seven representative geometries from an 80-subject dataset. To calculate Reynolds (Re) and Stokes (Stk) numbers we used the length characteristics of the idealized model reported by Grgic et al.^46^ for the adult subject and a 0.62 scaled version of that, as reported by Golshani et al.^47^ and Ruzycki et al.^48^, for the child subject. Calculated from the average flow rate data, $\hbox {Re}= 988$ and $\hbox {Stk}= 9.3E-4$ ($d_p =$ $3\upmu$m) and $2.6E-3$ ($d_p =$ $5\upmu$m), approximately $0.24\%$ ($d_p =$ $3\upmu$m) and $1.69\%$ ($d_p =$ $5\upmu$m) of particles deposit in the adult’s mouth-throat region. Deposition efficiencies are larger for the child subject, $1.69\%$ ($d_p = 3\upmu$m) and $6.75\%$ ($d_p = 5\upmu$m) for corresponding $\hbox {Re}= 862$ and $\hbox {Stk}= 2.1E-3$ ($d_p = 3\upmu$m) and $5.8E-3$ ($d_p = 5\upmu$m). For the infant, the predictive equations from Javaheri et al.^49^ was employed for a hydraulic diameter of 4.8 mm. As Re (1143) and Stk ($d_p =3$ $\upmu$m: 0.022 and $d_p = 5 \upmu$m: 0.059) numbers were larger in the infant, compared to the adult, more particles will likely be lost to the extra-thoracic airways for the infant; $2.8\%$ ($d_p = 3\upmu$m) and $7.5\%$ ($d_p = 5\upmu$m). Therefore, future work should be focused on including extra-thoracic airways, especially for infants. Note, that in the case of general anesthesia or severe lung injury endotracheal tubes may be used. The endotracheal tube will bypass the mouth and throat to control the respiratory cycle and will allow drugs to be released into the trachea. These intubations may be required in both adult and children and has been employed for those suffering from severe form of the COVID-19 disease^50^.

**Figure 7:**
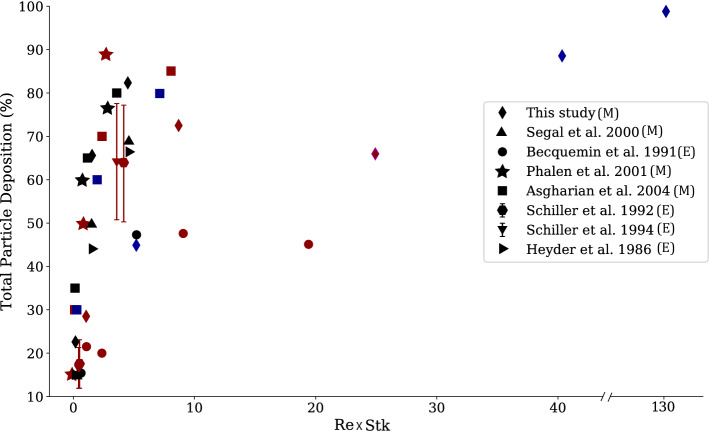
Total deposited dose for 1 $\upmu$m, 3 $\upmu$m, and 5 $\upmu$m diameter particles, with respect to Reynolds number ($Re = \frac{4 \rho Q_{mean}}{\pi \upmu D_{trachea}}$) times Stokes number ($Stk = \frac{\rho _p d_p^2 w}{18 \upmu d_c}$, where $d_p$ is the particle diameter, $\rho _p$ is the particle density, w the mean flow velocity, and $d_c$ the diameter of the airway), in both modeling (M) and experimental (E) studies. Infants are shown as blue, child as red, and adult subjects as black. Details on $Re\times Stk$ for each study are provided within the supplementary material.

We compared our calculations with experimental^51–54^ and computational^24,55^ data from the literature in terms of non-dimensional numbers ($Re\times Stk$, Fig. 7), only including studies that either measured or modeled deposition in the entire lung. Note, that for the experimental studies that did not include the trachea’s diameter, we used relationships provided in Phalen et al.^56^ to estimate it, based on the subject’s height and age. In general, our results agree with previous observations that deposition fractions are larger in infants and children, compared to adults^19,31^. Favorable comparisons are found despite the use of different mathematical and computational models. Similar to our observations, Hofmann et al.^57^ showed that peripheral airway dosimetry increases as age increases, as fewer particles deposit on the large conducting airways. Additionally, Heyder et al.^52^ measured regional deposition in adults ($d_p = 3\upmu$m: $Dep_C = 4\%$ and $Dep_R = 32\%$; $d_p = 1\upmu$m: $Dep_C = 0\%$ and $Dep_R = 15\%$). While our estimates are larger ($d_p = 3\upmu$m: $Dep_C = 18\%$ and $Dep_R = 47\%$; $d_p = 1\upmu$m: $Dep_C = 5\%$ and $Dep_R = 17\%$), the relationship between peripheral and distal deposition holds. Employing their lung dosimetry framework and assuming deposition efficiency is the same between inhalation and exhalation, Asgharian et al.^21^ predicted smaller deposition percentages in their 3-month infant, compared to ours (Fig. 7). Differences are likely due to airway size; the trachea diameter of Asgharian et al.^21^ was 7 mm, compared to our 4 mm.

While most studies show greater deposition fractions in infants and children compared to adults, some show the opposite for small particle sizes. For example, the modeling study by Phalen and Oldham found that children and adults have the same deposition percentages for $1\upmu$m diameter particles ($15\%$) at low physical activity levels^24^. They assumed 2.75 L/min and 10 L/min minute ventilations for the children and adult subjects, respectively, where we employed 8.1 L/min (child) and 15 L/min (adult). Our simulations estimate slightly larger deposition percentages (child: 28.5%; adult: 22.6%) for $1\upmu$m diameter particles. Indeed, using smaller flowrates should result in smaller deposition percentages within the respiratory track (as Reynolds and Stokes numbers would also be smaller). In contrast to our findings, Becquemin et al. also found similar deposition percentages in children and adults at rest^51^ ($20\%$ for $d_p = 1\upmu$m and $47\%$ for $d_p = 3\upmu$m, for tidal volumes of 400 mL (child) and 500 mL (adult). Differences may be attributed to subject size and respiration maneuver.

Modeling deposition throughout inhalation in the mouth-throat region and conducting airways for an adult, child, and infant subjects, Das et al.^29^ found $50\%$, $20\%$, and $15\%$ conducting airways deposition for $d_p = 3\upmu$m. This is in a good agreement with our predictions for inhalation ($65\%$, $23\%$, and $17\%$), Fig. 3. These findings suggest that simpler models (grouped whole lung models^24,57^ or models of inspiration only^29^) may provide similar insight into dosimetry as the models presented here, if hotspots or deposition throughout the entire respiration cycle are not needed.

To the best of our knowledge this is the first study to estimate deposited aerosol dose in realistic infant and child lungs, incorporating transport throughout the entire lung and respiration cycle. However, there are some limitations in this work that need to be acknowledged. Here, we only have three age groups and only one subject per group. Therefore, intersubject variability for each age group is not accounted for and we have yet to investigate the influence of demographics on dosimetry. In addition, by only including three ages, we cannot create a correlation model relating dose with age or lung volume. Due to the nature of the 1D modeling, all distal airways within a generation are grouped together and while variability in distal airways dimensions are taken into account by the diffusion coefficient and lobe-specific dimensions, we are still missing the actual variability in morphology. We assume that the conducting airway walls are rigid and respiratory airway walls deform linearly within the 1D model^32^ and that lobe volume fractions for all three age groups are the same as typical values^42^. In addition, we assume that the size or shape of the particles do not change during the time of simulation which may not be true for liquid droplets or nebulizers with high dose fractions^58,59^.

## Conclusion

Limited clinical trials on infants and children result in lack of evidence to properly choose the dosage and the device suitable for these young patients. Typically, clinicians estimate the dose needed for children based on data gathered from studies on adults by scaling the lung volume. Here, we built realistic airway geometries to study the lung dosimetry in an infant, child and adult throughout the whole breathing cycle. Our results highlight a larger deposition fraction in the infant subject compared to that of the child, which itself is higher than in the adult subject, suggesting that dosimetry in young subjects cannot be estimated from adults using only lung volumes or surface areas. This finding may provide guidance in development or improvement of public health policies regarding exposure limits. This study takes the first steps towards uncovering the dosimetry differences between each age group and to ultimately predict the dose needed for each young patient based on its lung structure to maximize the efficiency of the treatment while minimizing the side effects.

## Supplementary Information

Supplementary Information 1.

Supplementary Information 2.

Supplementary Information 3.

Supplementary Information 4.

Supplementary Information 5.
