# Supplementary material for: A whole lung in silico model to estimate age dependent particle dosimetry
Source: Sci Rep. 2021 May 27;11:11180. doi: 10.1038/s41598-021-90509-8 (PMC8159973; doi:10.1038/s41598-021-90509-8)
Supplement: Supplementary file 4 — Supplementary Information 4. [file 41598_2021_90509_MOESM4_ESM.pdf]

A Whole Lung *In Silico* Model to Estimate Age Dependent Particle Dosimetry

Kamran Poorbahrami[*], Irene E. Vignon-Clementel[†]

Shawn C. Shadden[‡], Jessica M. Oakes[§]

August 25, 2020

[*]Department of Mechanical and Industrial Engineering, Northeastern University, USA
[†]INRIA Paris, France
[‡]Department of Mechanical Engineering, University of California Berkeley, USA
[§]Department of Bioengineering, Northeastern University, USA

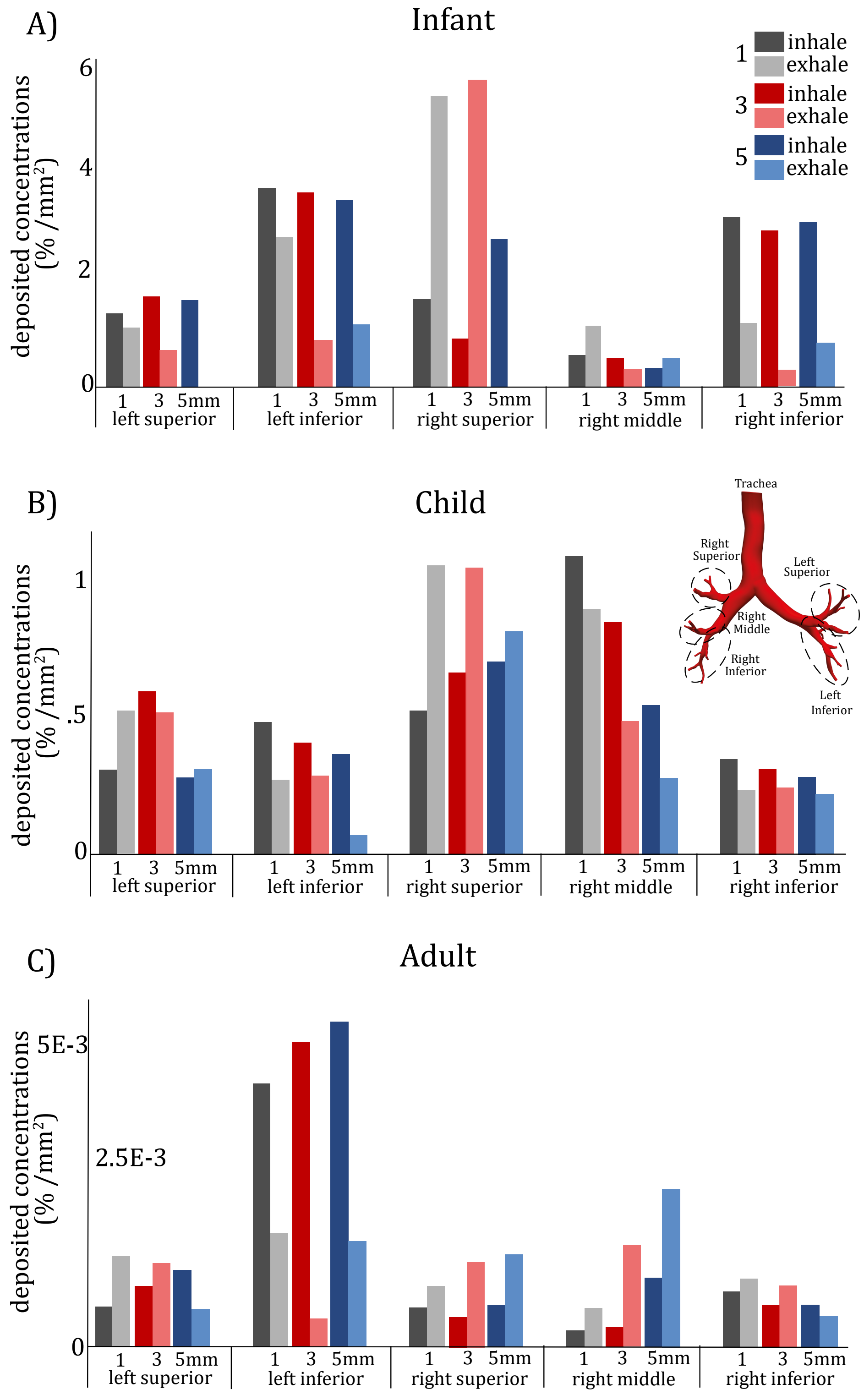

Figure 1S: Percent of particles that deposited on the airway walls of the 3D geometries, normalized the airway's respective surface areas. Results for both inhalation and exhalation are shown, represented by darker and lighter shades, respectively.
